# Adjunctive Non-Disease-Modifying Therapies in Multiple Sclerosis: Immunometabolic, Neuroprotective and Remyelination-Oriented Approaches

**DOI:** 10.3390/ijms27093857

**Published:** 2026-04-27

**Authors:** Agnieszka Damiza-Detmer, Andrzej Głąbiński

**Affiliations:** Department of Neurology and Stroke, Medical University of Lodz, Ul. Zeromskiego 113, 90-549 Lodz, Poland

**Keywords:** multiple sclerosis, adjunctive therapy, statins, metformin, immunometabolism, neuroprotection, remyelination, disease-modifying therapies

## Abstract

Disease-modifying therapies (DMTs) are the standard treatment for multiple sclerosis (MS) and effectively reduce inflammatory disease activity; however, their effects on neurodegeneration, remyelination failure, and long-term symptom burden vary across disease stages and patient populations. Adjunctive non-DMT approaches have therefore been investigated to target biological processes not primarily addressed by conventional immunomodulatory treatment. These strategies often involve agents originally developed for non-neurological indications, whose mechanisms of action extend beyond their primary clinical use and may intersect with pathways relevant to MS pathophysiology. This narrative review summarizes pharmacological agents, nutraceuticals, and selected bioactive compounds evaluated as adjunctive interventions in MS, with emphasis on immunometabolic regulation and remyelination-related mechanisms. Evidence from experimental models, translational studies, and clinical trials is examined to assess repurposed drugs and metabolic modulators with reported effects on immune responses, glial function, and myelin repair. Particular emphasis is placed on the distinction between mechanistic rationale and clinical outcomes, highlighting the challenges of translating biologically plausible effects into consistent therapeutic benefit. Available data indicate that most adjunctive strategies do not demonstrate consistent disease-modifying effects, although some interventions influence specific biological pathways relevant to neuroinflammation, cellular metabolism, and oligodendrocyte biology. Reported outcomes vary according to disease stage, treatment duration, and selected clinical or imaging endpoints. Overall, adjunctive non-DMT strategies remain investigational and require further evaluation in biologically stratified clinical studies.

## 1. Introduction

MS is a chronic immune-mediated disorder of the central nervous system (CNS) characterized by inflammatory demyelination, axonal injury, and neurodegenerative processes leading to progressive physical and cognitive disability. The disease comprises several clinical phenotypes, including relapsing-remitting MS (RRMS), secondary progressive MS (SPMS), primary progressive MS (PPMS), and relapsing-progressive MS (RPMS), which differ in disease course and patterns of disability accumulation. RRMS is defined by relapses followed by partial or complete recovery, whereas PPMS presents with progressive disability from onset without distinct relapses. SPMS represents a stage of transition from RRMS to relapse-independent disability progression [1].

In addition to clinical heterogeneity, MS is characterized by substantial biological and pathological variability across disease stages. Early inflammatory activity is typically associated with focal demyelinating lesions and blood-brain barrier (BBB) disruption, whereas later disease phases are increasingly dominated by neurodegenerative processes involving axonal loss, mitochondrial dysfunction, and impaired neuronal energy metabolism [2,3]. These mechanisms contribute to progressive disability accumulation even in the absence of overt inflammatory activity, suggesting that immune-mediated injury represents only one component of a broader and multifactorial disease process.

The development of DMTs has substantially improved control of inflammatory disease activity by reducing relapse rates and new lesion formation. However, clinical outcomes remain heterogeneous, and disability accumulation continues in a proportion of patients, particularly in progressive phenotypes where therapeutic options are limited [1]. Randomized clinical trial data in SPMS indicate that suppression of inflammatory activity alone may be insufficient to fully address disability progression, highlighting the contribution of additional pathological mechanisms [4].

An important contributor to long-term disability is the failure of efficient remyelination following demyelinating injury. Although the CNS retains a capacity for endogenous myelin repair through activation and differentiation of oligodendrocyte precursor cells (OPCs), this regenerative process becomes progressively less effective with disease duration and aging [2]. Experimental and neuropathological studies indicate that successful remyelination supports axonal integrity by restoring metabolic coupling between oligodendrocytes and neurons, as well as by re-establishing saltatory conduction along demyelinated axons [3]. Conversely, persistent demyelination exposes axons to increased metabolic stress, contributing to mitochondrial dysfunction, ion imbalance, and ultimately axonal degeneration.

Beyond focal inflammatory lesions, MS pathology involves interactions between immune cells, glial populations, and components of the neurovascular unit, including endothelial cells, pericytes, astrocytic endfeet, and basement membrane structures that regulate CNS homeostasis and barrier function [5,6]. Disruption of these interactions contributes to sustained tissue injury and impaired repair mechanisms. Persistent microglial activation, metabolic dysregulation, oxidative stress, and oligodendrocyte dysfunction have been implicated in neurodegeneration and limited endogenous remyelination, independent of overt inflammatory activity [5,7].

In parallel, increasing attention has been directed toward metabolic pathways that influence oligodendrocyte function and regenerative capacity. Experimental studies suggest that systemic metabolic factors can modulate remyelination efficiency by affecting OPC differentiation, cellular bioenergetics, and inflammatory signaling pathways. For example, metabolic interventions targeting mitochondrial function and cellular energy-sensing pathways have been shown to restore the regenerative potential of aged OPC populations in experimental models, highlighting a potential link between metabolic regulation and myelin repair [8]. These findings support the concept that immunometabolic mechanisms may represent an important therapeutic target beyond classical immunomodulation.

Given the heterogeneity of long-term outcomes under DMTs, therapeutic strategies not primarily intended to modify inflammatory disease activity have been explored as adjunctive approaches in MS. These include repurposed pharmacological agents, metabolic modulators, nutraceuticals, and selected bioactive compounds evaluated alongside standard therapies [9]. Importantly, many of these interventions were originally developed for indications unrelated to MS, yet their mechanisms of action intersect with pathways implicated in neuroinflammation, cellular metabolism, and oligodendrocyte biology.

This aspect is particularly relevant in the context of comorbid conditions frequently observed in MS, including metabolic syndrome, obesity, dyslipidemia, and diabetes, which have been associated with disease severity and progression [10,11]. In such settings, pharmacological agents targeting systemic metabolic pathways may exert biological effects that extend beyond their primary indications and interact with mechanisms involved in neuroinflammation, cellular metabolism, and tissue repair. This overlap also reflects the concept of pharmacological repurposing, in which agents used for common comorbid conditions may exert parallel effects on MS-related biological pathways, representing a potential, although not yet clinically validated, dimension of individualized therapeutic strategies.

Such interventions have been investigated for their reported effects on immune regulation, cellular metabolism, glial activation, and oxidative stress—processes not fully addressed by conventional immunomodulatory treatment [5]. Clinical evidence remains variable and dependent on disease stage, study design, and endpoint selection, underscoring the complexity of interpreting adjunctive therapy effects in MS [4,12].

Consequently, adjunctive non-DMT approaches are increasingly investigated as complementary strategies aimed at targeting biological pathways associated with neurodegeneration, metabolic dysfunction, and impaired remyelination. Rather than replacing established DMTs, these interventions are primarily intended to modulate specific aspects of MS pathophysiology that remain insufficiently addressed by current immunomodulatory treatments.

In this context, adjunctive strategies can be conceptually organized into approaches targeting immunometabolic dysregulation, modulation of oxidative and cellular stress pathways, and promotion of endogenous remyelination, thereby reflecting a shift from purely anti-inflammatory paradigms toward broader regulation of CNS homeostasis and repair mechanisms.

The present review critically summarizes mechanistic and clinical data on adjunctive non-DMT strategies, with emphasis on immunometabolic modulation and remyelination-related interventions, while distinguishing preclinical findings from clinical outcomes.

**Figure 1:**
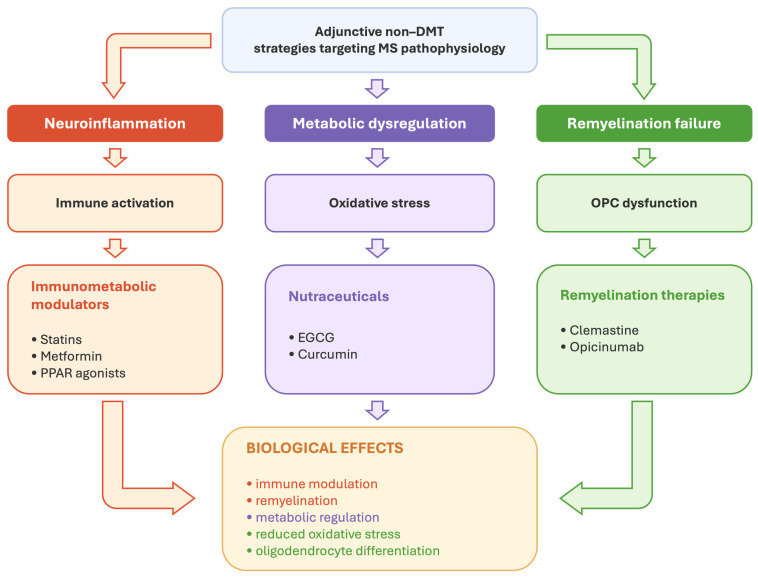
Conceptual Framework of Adjunctive Non-DMT Strategies in Multiple Sclerosis. MS pathology involves interacting processes, including neuroinflammation, metabolic dysregulation, and failure of endogenous remyelination. Adjunctive non-disease-modifying therapies investigated in MS target these mechanisms through immunometabolic modulation (e.g., statins, metformin, PPAR agonists), nutraceutical and bioactive compounds (e.g., epigallocatechin-3-gallate and curcumin (EGCG)), and remyelination-oriented therapies (e.g., clemastine and anti-LINGO-1 antibody opicinumab). These approaches may influence immune signaling, cellular metabolism, oxidative stress pathways, and oligodendrocyte lineage biology.

The conceptual framework of adjunctive non-disease-modifying strategies in multiple sclerosis is summarized in Figure 1.

## 2. Immunometabolic Modulation as an Adjunctive Therapeutic Strategy in MS

Immunometabolic dysregulation contributes to chronic inflammation, neurodegeneration, and impaired repair mechanisms in MS, extending beyond pathways directly targeted by DMTs. Alterations in cellular energy sensing, lipid metabolism, and mitochondrial function influence immune cell activation, glial responses, and CNS tissue homeostasis, thereby affecting disease progression and symptom persistence [5,6]. These processes are increasingly recognized in both experimental and translational studies of MS.

Immunometabolism describes the reciprocal relationship between cellular metabolic pathways and immune cell function, in which metabolic states directly influence immune activation, differentiation, and effector responses. In the context of chronic neuroinflammatory disorders such as MS, metabolic reprogramming of immune and glial cells contributes to sustained inflammatory signaling, oxidative stress, and impaired regenerative processes within the CNS [5,13]. Activated immune cells frequently shift toward glycolysis-dependent energy production, whereas regulatory and reparative cell phenotypes are more strongly associated with mitochondrial oxidative phosphorylation and lipid metabolism [13,14]. These metabolic shifts can influence microglial activation states, astrocytic responses, and oligodendrocyte lineage dynamics, thereby linking systemic metabolic status with local CNS pathology.

Pharmacological agents and bioactive compounds with immunometabolic activity have therefore been investigated as adjunctive interventions, primarily for their effects on metabolic regulation, oxidative stress pathways, and glial function rather than direct suppression of inflammatory disease activity [9]. Mechanistic studies further support the role of metabolic pathways in shaping immune and glial phenotypes relevant to chronic CNS injury [13,15].

In addition to immune regulation, metabolic signaling pathways are increasingly recognized as modulators of endogenous repair mechanisms, including OPC activation and differentiation. Experimental data suggest that metabolic stress, mitochondrial dysfunction, and lipid dysregulation can impair remyelination capacity by altering the regenerative potential of oligodendrocyte lineage cells [2]. Consequently, therapeutic strategies targeting metabolic pathways may influence not only inflammatory responses but also processes involved in myelin repair and neuroprotection.

Statins and metformin represent the most extensively studied immunometabolic modulators in MS. Statins have been evaluated for immunomodulatory effects beyond lipid-lowering, whereas metformin has been investigated for its role in modulating cellular energy-sensing pathways in experimental and clinical settings [16,17,18,19].

Additional metabolic regulators, including ligands of peroxisome proliferator-activated receptors (PPARs), have also been explored due to their roles in lipid metabolism, mitochondrial function, and transcriptional control of inflammatory pathways [20,21]. Together, these agents illustrate the broader concept of immunometabolic modulation as an adjunctive therapeutic strategy targeting biological mechanisms that extend beyond classical immunomodulation.

### 2.1. Statin

Statins have been investigated in MS as adjunctive agents in relation to their reported immunomodulatory and cellular effects that extend beyond lipid-lowering.

At the biochemical level, statins inhibit 3-hydroxy-3-methylglutaryl-coenzyme A (HMG-CoA) reductase, a key enzyme of the mevalonate pathway responsible for cholesterol synthesis and the generation of isoprenoid intermediates involved in intracellular signaling. Inhibition of this pathway affects the prenylation of small GTP-binding proteins such as Ras and Rho, which regulate immune cell activation, migration, and cytokine production [17,22]. Through these mechanisms, statins may influence both peripheral immune responses and inflammatory signaling pathways relevant to autoimmune CNS injury.

In experimental autoimmune encephalomyelitis (EAE), atorvastatin administration was associated with changes in T-cell polarization, characterized by promotion of T helper 2 (Th2) responses, reflected by increased secretion of interleukin-4 (IL-4), IL-5, IL-10, and transforming growth factor-β (TGF-β), together with concomitant suppression of T helper 1 (Th1) responses, including reduced production of IL-2, IL-12, interferon-γ (IFN-γ), and tumor necrosis factor-α (TNF-α) [17]. These immunological changes were accompanied by attenuated clinical disease severity and reduced CNS inflammatory infiltration in the EAE model.

Statins have also been shown to interfere with leukocyte adhesion through inhibition of leukocyte function antigen-1 (LFA-1)-dependent interactions, limiting leukocyte activation and transendothelial migration [22].

Beyond peripheral immune modulation, statins may also influence CNS-resident cell populations involved in neuroinflammation and tissue repair. Experimental evidence suggests that statins can modulate microglial activation and inflammatory mediator production, indicating potential effects on the neuroimmune environment within demyelinating lesions [23]. Moreover, modulation of isoprenoid-dependent signaling pathways has been implicated in cellular processes relevant to oligodendrocyte survival and differentiation, although the precise relevance of these mechanisms in MS pathology remains incompletely understood. However, experimental data also indicate that statins may exert context-dependent effects on oligodendrocyte biology, including inhibition of oligodendrocyte progenitor differentiation and impairment of remyelination under certain conditions [24]. This highlights the complexity of targeting pathways that may simultaneously modulate immune responses and intrinsic CNS repair processes. These signaling pathways converge on processes regulating oligodendrocyte cytoskeletal dynamics, membrane synthesis, and myelin formation, which are essential for effective remyelination and maintenance of axonal integrity.

Experimental studies have also reported that simvastatin has been reported to influence oligodendroglial process dynamics and cell survival under experimental conditions [23]. These findings indicate effects on both peripheral immune responses and CNS-resident cells. However, modulation of prenylation-dependent pathways may exert context-dependent effects, as interference with Ras and Rho signaling has also been associated with impaired oligodendrocyte process extension and myelin formation under certain experimental conditions [23].

Collectively, preclinical studies demonstrate multiple biological actions of statins relevant to immune regulation and CNS cellular function. However, translation of these mechanisms into clinical benefit has been variable and dependent on disease stage and outcome selection.

#### Statins—Evidence from Clinical Studies

Clinical evaluation of statins in MS has included interventional and observational studies with heterogeneous outcomes across disease phenotypes and endpoints. In SPMS, simvastatin treatment was associated with a reduced annualized brain atrophy rate, while effects on disability-related outcomes were modest and not consistently correlated with inflammatory activity [25]. This randomized placebo-controlled trial demonstrated a reduction in whole-brain atrophy over a two-year treatment period, suggesting that statins may exert effects on neurodegenerative processes that are at least partly independent of classical anti-inflammatory mechanisms. Subsequent data examining disability progression as a primary endpoint reported limited clinical effects despite biological signals suggestive of non-inflammatory mechanisms [4].

The potential relevance of statins for progressive MS has been discussed in the context of mechanisms linked to neurodegeneration, mitochondrial dysfunction, and impaired remyelination. Experimental and translational studies indicate that modulation of the mevalonate pathway may influence cellular processes involved in axonal maintenance, oxidative stress responses, and glial activation, providing a biological rationale for evaluating statins in progressive disease phenotypes where inflammatory activity is less prominent [12,26].

Observational studies assessing statin exposure and disability outcomes have yielded variable results. In a population-based cohort, statin use was not consistently associated with reduced disability progression, highlighting potential confounding factors including indication bias and disease phenotype [27].

Additional observational analyses examining MRI and clinical outcomes have similarly reported heterogeneous findings. Some studies suggest modest associations with imaging markers such as brain atrophy or lesion burden, whereas others do not demonstrate measurable effects on disability accumulation. These discrepancies likely reflect differences in study design, disease stage, treatment duration, and concomitant DMT use [12,27].

Systematic reviews integrating available studies conclude that statins do not demonstrate consistent disease-modifying effects in MS, although effects on selected imaging or secondary outcomes have been reported in specific contexts [12,26].

Overall, clinical findings vary according to disease stage, study design, and endpoint selection. Current evidence therefore suggests that while statins exhibit measurable biological activity relevant to MS pathophysiology, their clinical benefits remain uncertain and appear limited to selected outcomes or patient subgroups rather than consistent modification of disease progression.

### 2.2. Metformin

Metformin has been investigated in MS as an adjunctive intervention primarily in the context of immunometabolic regulation rather than direct modulation of inflammatory disease activity. Originally developed as an antidiabetic agent, metformin has attracted increasing interest in neurological disorders due to its effects on cellular metabolism, mitochondrial function, and inflammatory signalling pathways. These properties have led to the investigation of metformin in diseases characterized by metabolic dysregulation and impaired regenerative capacity, including MS.

At the molecular level, metformin is known to activate adenosine monophosphate-activated protein kinase (AMPK), a central regulator of cellular energy homeostasis that integrates metabolic status with inflammatory and stress-related signalling pathways [13]. AMPK activation modulates immune cell function, reduces pro-inflammatory signalling, and influences cellular metabolic programs involved in immune activation. AMPK activation influences multiple downstream pathways involved in cellular metabolism, including regulation of mitochondrial activity, lipid metabolism, and transcriptional programs controlling immune activation and cellular stress responses. Through these mechanisms, metformin may influence both peripheral immune responses and metabolic processes within CNS-resident cells [13,18]. In addition to AMPK activation, metformin has been shown to modulate mitochondrial complex I activity, redox-sensitive signaling pathways, and mTOR-dependent processes, indicating that its biological effects extend beyond a single signaling axis [13,15]. These mechanisms may collectively influence cellular energy balance, oxidative stress responses, and metabolic adaptation in both immune and glial cells. These observations indicate that metformin’s biological effects cannot be attributed solely to AMPK activation but rather reflect the integration of multiple metabolic and signaling pathways.

Metabolic regulation has emerged as an important determinant of immune cell activation and glial responses in neuroinflammatory disorders. Experimental and translational studies indicate that metabolic pathways influence microglial activation states, inflammatory mediator production, and cellular responses to tissue injury [13,15]. These findings provide a mechanistic rationale for investigating metabolic modulators such as metformin as adjunctive therapeutic strategies in MS.

Experimental data indicate that AMPK activation is associated with reduced nuclear factor-κB (NF-κB)-dependent transcription and attenuation of neuroinflammatory responses in models of immune-mediated demyelination. In experimental autoimmune encephalomyelitis (EAE), metformin administration has been associated with reduced disease severity and decreased expression of pro-inflammatory mediators within the CNS, suggesting that metabolic regulation can modulate neuroinflammatory processes in demyelinating disease models [28,29].

Beyond immune modulation, increasing attention has focused on the potential role of metformin in regulating oligodendrocyte lineage biology and remyelination. These metabolic pathways converge on processes regulating oligodendrocyte precursor cell differentiation, mitochondrial function, and lipid biosynthesis, which are essential for myelin membrane formation and long-term axonal support [8,30]. Oligodendrocyte precursor cells undergo substantial metabolic transitions during differentiation and myelin synthesis, processes that require coordinated regulation of mitochondrial activity and lipid biosynthesis. Disruption of these pathways has been implicated in impaired remyelination observed in chronic demyelinating conditions [7,8].

Moreover, metformin has been reported to influence oligodendrocyte lineage cells and myelin repair processes under experimental conditions, including enhanced differentiation capacity of oligodendrocyte precursor cells, particularly in aging-related contexts. Experimental studies have demonstrated that metformin can restore the regenerative potential of aged oligodendrocyte progenitor cells and improve remyelination efficiency following demyelinating injury, suggesting that metabolic interventions may partially reverse age-associated deficits in myelin repair [8]. This effect has been linked to restoration of metabolic competence in oligodendrocyte lineage cells, suggesting that metabolic interventions may directly influence the capacity for remyelination and preservation of axonal integrity in demyelinating conditions [8].

Recent work using human oligodendrocyte lineage cells has further highlighted the importance of age-dependent cellular responses to metformin. In vitro studies demonstrated that metformin enhances the ensheathment capacity of adult human oligodendrocyte progenitors while producing different responses in pediatric-derived cells, indicating developmental stage-dependent metabolic regulation of oligodendrocyte function [7].

Further experimental data indicate that metformin may influence mitochondrial metabolism and lipid biosynthetic pathways that are essential for myelin membrane formation. Modulation of these metabolic processes has been proposed as one of the mechanisms through which metformin may support oligodendrocyte differentiation and remyelination in demyelinating disease models [30].

Clinical investigations in MS have primarily focused on biological and imaging endpoints rather than disease-modifying efficacy. Available clinical studies have largely evaluated biomarker changes, metabolic parameters, or exploratory imaging outcomes rather than relapse activity or long-term disability progression.

Available studies report modulation of metabolic and inflammatory markers, with variable effects depending on disease stage and study design [9,15]. Small clinical and translational studies have reported alterations in inflammatory mediators or metabolic markers, although consistent effects on relapse activity or disability progression have not been demonstrated [19,31,32,33,34].

Overall, current data do not demonstrate consistent effects on relapse activity or disability progression in MS. Taken together, existing evidence indicates that metformin exerts measurable biological effects on immunometabolic pathways and oligodendrocyte lineage biology, but its clinical efficacy in modifying disease progression in MS remains unconfirmed. Its potential role is therefore primarily considered within the framework of adjunctive strategies targeting metabolic regulation and remyelination-related processes.

#### 2.2.1. Preclinical Evidence

Preclinical studies have evaluated metformin in models of immune-mediated demyelination, primarily EAE. These experimental systems have provided important insights into the potential immunometabolic and neuroprotective actions of metformin in demyelinating disease.

In EAE, metformin administration was associated with reduced clinical severity and decreased inflammatory activity, interpreted in the context of AMPK-dependent metabolic regulation [28,29]. Treatment was accompanied by reduced infiltration of inflammatory cells into the CNS and decreased production of pro-inflammatory cytokines, suggesting that modulation of cellular metabolism can influence immune-mediated tissue injury in demyelinating models. These effects included reduced expression of pro-inflammatory mediators within the CNS.

Additional studies indicate that metformin modulates microglial activation and inflammatory signaling pathways involved in neuroinflammation. Experimental data suggest that metformin can influence microglial polarization and attenuate inflammatory signalling pathways associated with chronic neuroinflammation, further supporting the concept that metabolic pathways regulate glial responses in demyelinating disease models. Such changes in microglial activation states may further contribute to a microenvironment that supports oligodendrocyte differentiation and limits secondary axonal damage in demyelinating lesions [28,35].

Beyond immune modulation, metformin has been reported to influence oligodendrocyte lineage dynamics, including enhanced differentiation capacity of oligodendrocyte precursor cells and improved remyelination efficiency under aging-related conditions [8]. These findings suggest that metabolic interventions may partially reverse age-associated impairments in oligodendrocyte regenerative capacity, which has been proposed as one of the factors limiting remyelination in progressive stages of demyelinating disease.

More recent studies using human oligodendrocyte lineage cells have further demonstrated that metformin can influence myelin-related cellular functions, including the capacity of oligodendrocyte progenitors to extend processes and ensheath axon-like structures in vitro [30]. Experimental observations also suggest that metformin-induced metabolic changes may affect mitochondrial activity and lipid biosynthesis pathways required for myelin membrane formation [30].

Collectively, preclinical evidence supports AMPK-dependent effects of metformin on immune regulation, glial activation, and oligodendrocyte biology in EAE models. However, these findings derive from controlled experimental systems and require cautious interpretation in relation to human MS.

#### 2.2.2. Clinical Studies

Clinical investigations of metformin in MS have primarily evaluated biological, imaging, and exploratory clinical endpoints rather than disease-modifying efficacy. Compared with the relatively extensive experimental literature, clinical evidence remains limited and heterogeneous.

Early clinical data reported acceptable tolerability and modulation of metabolic and inflammatory parameters without significant effects on disability outcomes [36]. These early studies primarily assessed safety and metabolic biomarkers rather than clinical efficacy endpoints.

Subsequent pilot and exploratory studies similarly observed changes in inflammatory or metabolic biomarkers, while effects on relapse activity or short-term disability progression were not consistently demonstrated [32,33,34]. Some investigations reported alterations in circulating inflammatory mediators or metabolic markers, suggesting biological activity of metformin in MS patients, although these findings were not consistently associated with measurable clinical benefits.

Observational analyses have further examined imaging and biomarker-based outcomes, with findings varying according to disease stage and study design [37]. Differences in patient populations, treatment duration, concomitant DMT use, and outcome measures may contribute to variability observed across studies.

Available clinical data do not demonstrate consistent effects of metformin on relapse rate or disability progression in MS. Consequently, metformin is currently not considered a DMT in MS but remains of interest as a potential adjunctive agent targeting metabolic pathways implicated in neuroinflammation and impaired remyelination.

Current evidence supports biological activity but remains insufficient to establish clinical efficacy.

### 2.3. PPARs as Immunometabolic Modulators

PPARs are ligand-activated nuclear receptors that regulate lipid metabolism, glucose homeostasis, and inflammatory signaling through transcriptional control of metabolic and immune-related genes. In MS, PPAR activation has been investigated as an adjunctive strategy targeting immunometabolic pathways rather than direct suppression of inflammatory disease activity [21].

PPARs belong to the nuclear receptor superfamily and function as transcription factors that heterodimerize with retinoid X receptors (RXRs), enabling regulation of genes involved in lipid metabolism, mitochondrial function, and inflammatory responses [38,39]. Three major isoforms—PPAR-α, PPAR-β/δ, and PPAR-γ—have been identified, each displaying distinct tissue distribution and metabolic roles. Importantly, the biological effects of PPAR activation are highly isoform-specific and context-dependent, varying across immune cell subsets, glial populations, and metabolic conditions. These receptors integrate metabolic signals with immune regulation, thereby linking systemic metabolic status with inflammatory processes relevant to chronic neurological disease [38,39,40].

Preclinical studies indicate that activation of PPAR-α and PPAR-γ modulates immune cell function, reduces pro-inflammatory cytokine production, and alters microglial activation states in models of demyelination [20,39]. Experimental evidence further suggests that PPAR signaling can influence the balance between pro-inflammatory and anti-inflammatory immune responses through transcriptional repression of inflammatory mediators and modulation of macrophage and microglial polarization [38,41].

PPAR signaling has also been linked to regulation of mitochondrial function and lipid metabolism within CNS-resident cells, processes implicated in chronic tissue injury [40,41]. Since myelin membranes contain exceptionally high lipid content, pathways regulating lipid biosynthesis and mitochondrial energy metabolism are particularly relevant for oligodendrocyte function and myelin maintenance [41]. These pathways may therefore converge on mechanisms regulating oligodendrocyte differentiation, myelin synthesis, and maintenance of axonal integrity [41,42]. Dysregulation of these metabolic processes has been proposed as one factor contributing to impaired remyelination in demyelinating disorders.

Recent studies further suggest that activation of PPAR pathways may influence oligodendrocyte lineage dynamics and neuroinflammatory signaling networks involved in CNS repair processes [42]. Experimental investigations indicate that PPAR signaling interacts with pathways regulating cellular metabolism, mitochondrial activity, and oxidative stress responses within glial cells [40,41,42]. These findings have contributed to growing interest in PPAR agonists as potential modulators of metabolic and inflammatory processes relevant to demyelinating disease.

Despite these mechanistic observations, clinical evidence supporting PPAR agonists in MS remains limited. The lack of consistent translation may reflect differences in isoform targeting, systemic metabolic effects, and challenges in identifying patient subgroups most likely to benefit from immunometabolic modulation [21]. Most available data derive from experimental or translational studies, and robust clinical trials assessing disability or disease activity endpoints are lacking [21]. Accordingly, PPAR-targeting strategies are currently considered exploratory adjunctive approaches rather than established disease-modifying interventions.

#### Fibrates

Fibrates are lipid-lowering agents acting primarily as PPAR-α agonists and have been explored in MS due to their immunometabolic effects. Fenofibrate, the most extensively studied compound in this class, has been evaluated for its influence on inflammatory signaling and lipid metabolism in immune and glial cells [43].

Through activation of PPAR-α, fibrates regulate transcription of genes involved in fatty acid oxidation, lipid transport, and mitochondrial metabolism [40,41]. These pathways are closely linked to cellular energy homeostasis and inflammatory signaling, providing a mechanistic rationale for investigating fibrates in inflammatory and neurodegenerative disorders.

Preclinical studies suggest that fenofibrate reduces pro-inflammatory cytokine production and oxidative stress through PPAR-α-dependent transcriptional regulation [44,45]. Experimental findings also indicate that PPAR-α activation may attenuate microglial activation and modulate inflammatory signaling pathways implicated in CNS tissue injury [41]

These findings provide a mechanistic rationale for investigation in demyelinating disease but derive predominantly from experimental settings. In addition to immunomodulatory effects, metabolic regulation through PPAR-α signaling may influence lipid metabolism pathways relevant to myelin membrane synthesis and maintenance, further supporting interest in fibrates as potential modulators of demyelinating disease mechanisms [40].

Clinical data on fibrate use in MS are sparse. Reviews of available evidence indicate that fibrates have not been systematically evaluated in randomized controlled trials in MS, and existing data are insufficient to determine their impact on disability progression or disease activity [21,43]. Consequently, fibrates are currently considered experimental adjunctive agents in MS, with their potential role supported mainly by mechanistic rationale rather than clinical efficacy data.

## 3. Nutraceuticals and Bioactive Compounds as Adjunctive Approaches in MS

### 3.1. Polyphenols with Immunometabolic and Neuroprotective Properties: Epigallocatechin-3-Gallate

Epigallocatechin-3-gallate (EGCG) is a polyphenolic compound derived from green tea that has been investigated in MS due to its reported immunometabolic and antioxidant properties. At the molecular level, EGCG interacts with multiple signaling pathways involved in inflammatory responses, oxidative stress regulation, and cellular metabolism [46].

EGCG belongs to the catechin family of polyphenols and exhibits pleiotropic biological activity, including modulation of intracellular signaling pathways, regulation of mitochondrial function, and antioxidant activity through scavenging of reactive oxygen species. These properties have attracted interest in neuroinflammatory and neurodegenerative disorders, where oxidative stress and mitochondrial dysfunction contribute to tissue injury [47].

Preclinical studies indicate that EGCG reduces pro-inflammatory cytokine production, modulates redox-sensitive signaling cascades, and influences glial cell responses in experimental models of demyelination [46,48]. These effects have been linked to the regulation of oxidative stress and intracellular signaling pathways implicated in neuroinflammation.

Studies in EAE demonstrated that EGCG can suppress autoreactive T-cell proliferation and reduce production of inflammatory mediators such as tumor necrosis factor-α. In addition, EGCG has been shown to interfere with proteasome activity and inhibit nuclear factor-κB activation, thereby attenuating inflammatory signaling pathways involved in immune-mediated CNS injury [49]

Additional experimental and translational data suggest that EGCG may influence lipid metabolism and cellular energy homeostasis, as well as stress responses in neuronal and oligodendrocyte populations [50]. These mechanisms are particularly relevant in demyelinating disorders, where oxidative stress and metabolic dysregulation may contribute to oligodendrocyte injury and impaired remyelination.

Clinical investigations in MS have yielded heterogeneous findings. Randomized and non-randomized studies have primarily assessed biological, imaging, and functional outcomes rather than disease-modifying efficacy. EGCG supplementation has been associated with changes in inflammatory and metabolic markers, while effects on disability progression and clinical endpoints have been inconsistent across studies [51,52].

Systematic reviews conclude that current evidence remains insufficient to establish a clinically meaningful effect on disease course [46].

Overall, EGCG represents a bioactive compound with mechanistic and translational relevance in MS; however, confirmation of clinical efficacy requires adequately powered trials with clearly defined endpoints.

### 3.2. Other Nutraceuticals and Herbal Compounds

In addition to polyphenols such as EGCG, various nutraceuticals and herbal compounds have been explored as adjunctive approaches in MS, primarily based on their reported immunomodulatory, antioxidant, and metabolic properties [53,54]. Unlike conventional pharmacological agents that target specific molecular pathways, many nutraceuticals exert pleiotropic effects by modulating multiple signaling cascades involved in immune regulation, oxidative stress responses, and cellular metabolism.

Mechanistic studies summarized in recent reviews indicate that several plant-derived compounds regulate inflammatory signaling via NF-κB-dependent transcription, mitogen-activated protein kinase (MAPK) pathways, and redox-sensitive cascades. In experimental models of neuroinflammation and demyelination, such modulation has been associated with reduced pro-inflammatory mediator production, altered microglial activation states, and regulation of mitochondrial function, suggesting potential effects on both immune responses and neuronal homeostasis [54,55].

Among nutraceutical compounds investigated in the context of MS, curcumin, a polyphenolic compound derived from Curcuma longa, has attracted considerable attention due to its broad immunometabolic and neuroprotective properties. Curcumin interacts with multiple molecular pathways involved in neuroinflammation and cellular metabolism, including NF-κB, PI3K/Akt/mTOR, AMPK, and PPAR-related signaling networks [56]. However, curcumin is characterized by poor bioavailability due to limited absorption, rapid metabolism, and low systemic stability, which significantly restricts its translational potential in clinical settings [56,57]. Through these mechanisms, curcumin has been reported to reduce the production of pro-inflammatory cytokines, inhibit microglial and astrocytic activation, and attenuate oxidative stress in models of CNS inflammation [55,57].

Further studies indicate that curcumin may influence mitochondrial function, autophagy, and apoptosis pathways that are implicated in neurodegenerative processes associated with MS. In animal models of EAE, curcumin administration has been associated with reduced inflammatory infiltration, decreased demyelination, and modulation of T-cell responses, including effects on Th1 and Th17 cytokine signaling [56,57]. Additional studies suggest that curcumin may also regulate oxidative stress-related pathways and enhance endogenous antioxidant defenses, potentially contributing to neuroprotective effects in inflammatory CNS conditions [55].

Despite these mechanistic findings, translation into clinical evidence remains limited. Small clinical studies evaluating nano-formulated curcumin or related delivery systems in MS have attempted to overcome these pharmacokinetic limitations; however, these approaches remain experimental, and their ability to achieve clinically meaningful therapeutic concentrations in the CNS has not been conclusively demonstrated. However, consistent or clinically meaningful improvements in relapse activity, disability progression, or long-term outcomes have not been demonstrated in MS populations [57]. These limitations, combined with variability in formulation and dosing strategies, further complicate interpretation of clinical outcomes and highlight the gap between mechanistic plausibility and therapeutic efficacy [56,57].

Traditional multi-component formulations, including preparations derived from Chinese herbal medicine, have also been investigated in experimental and limited clinical contexts. These formulations often combine multiple bioactive compounds that may influence immune activation, oxidative stress pathways, and metabolic signaling networks. However, variability in composition, dosing, and methodological design complicates reproducibility and mechanistic interpretation of these findings [58].

Overall, nutraceutical and herbal compounds represent biologically plausible adjunctive approaches in MS. Their effects appear to involve modulation of inflammatory signaling, oxidative stress pathways, and cellular metabolic processes. Nevertheless, current clinical evidence remains limited, and adequately powered trials are required to determine whether these biologically active compounds can translate into clinically meaningful therapeutic benefits [53,54,58].

## 4. Remyelination-Oriented Adjunctive Therapies

### 4.1. Antihistamines and Muscarinic Receptor Modulation: Clemastine Fumarate

Failure of endogenous remyelination contributes substantially to axonal degeneration and disability progression in MS, particularly in chronic disease stages. Increasing attention has been directed toward therapeutic strategies that promote oligodendrocyte lineage differentiation and myelin repair rather than directly suppressing inflammatory activity. Clemastine fumarate, a first-generation antihistamine with anticholinergic properties, has emerged as a candidate compound with remyelination-promoting potential [59,60,61].

Although originally developed as a histamine H1 receptor antagonist, the remyelination-related effects of clemastine appear to be mediated primarily through antagonism of muscarinic acetylcholine receptors, particularly the M1 subtype expressed on OPCs. Inhibition of M1 signaling promotes OPC differentiation and accelerates maturation into myelinating oligodendrocytes, thereby enhancing myelin formation in experimental models [62,63]. These findings emerged from high-throughput screening studies that identified clemastine as a compound capable of stimulating oligodendrocyte differentiation in vitro and promoting remyelination in animal models of demyelination.

Subsequent experimental work has further supported the capacity of clemastine to modulate oligodendroglial lineage dynamics and myelin repair processes. In models of toxin-induced demyelination, clemastine treatment has been associated with increased oligodendrocyte differentiation, accelerated remyelination, and improved conduction across previously demyelinated axons [63,64]. Additional studies have suggested that clemastine may also influence glial responses and neuronal survival pathways involved in demyelinating injury, although these mechanisms remain incompletely characterized [65].

Clinical evaluation of clemastine in MS has primarily focused on functional and electrophysiological markers of remyelination. In the randomized, double-blind, placebo-controlled ReBUILD trial involving patients with chronic optic neuropathy, treatment with clemastine was associated with a modest reduction in visual evoked potential (VEP) latency, interpreted as an indicator of improved conduction along demyelinated optic nerve fibers and therefore consistent with partial remyelination [59]. However, no significant changes were observed in relapse activity or disability progression over the study period.

More recent translational and clinical analyses have continued to explore the potential role of clemastine in remyelination-oriented therapeutic strategies. Emerging data suggest that antihistamine-mediated modulation of muscarinic signaling may influence oligodendrocyte lineage cell biology and myelin repair in the central nervous system, supporting continued interest in this pharmacological class as potential remyelinating agents [61,66]. Nevertheless, clinical effects reported to date remain modest, and the extent to which electrophysiological improvements translate into meaningful long-term neurological benefit remains uncertain.

Safety and tolerability considerations also remain relevant for clinical application. As a first-generation antihistamine with anticholinergic activity, clemastine is associated with adverse effects, including sedation and anticholinergic symptoms, which may limit its tolerability in some patients during prolonged treatment [60].

Overall, clemastine fumarate represents one of the most extensively studied pharmacological candidates targeting remyelination in MS. Current evidence supports its biological activity in promoting oligodendrocyte differentiation and enhancing remyelination in experimental systems, with preliminary clinical data indicating modest electrophysiological improvement. However, further studies are required to determine whether these mechanistic and functional effects translate into sustained clinical benefit in patients with MS.

### 4.2. Anti-LINGO-1 Therapy: Opicinumab

Opicinumab is a human monoclonal antibody targeting leucine-rich repeat and immunoglobulin-like domain-containing protein 1 (LINGO-1), a negative regulator of oligodendrocyte differentiation and myelination. Inhibition of LINGO-1 has been proposed to promote remyelination by enhancing oligodendrocyte progenitor cell maturation and myelin repair [67]. LINGO-1 is a transmembrane protein expressed in neurons and oligodendrocyte lineage cells and forms part of the Nogo receptor signaling complex, which negatively regulates axonal regeneration and oligodendrocyte differentiation. Through this signaling network, LINGO-1 limits myelin formation and contributes to impaired endogenous remyelination in demyelinating conditions [67,68].

Preclinical studies demonstrated increased oligodendrocyte differentiation and myelination following LINGO-1 blockade, providing a mechanistic rationale for clinical investigation. Experimental models of demyelination further showed that inhibition of LINGO-1 promotes maturation of oligodendrocyte precursor cells, enhances remyelination, and improves axonal conduction across previously demyelinated fibers, supporting the concept that antagonizing LINGO-1 may restore endogenous myelin repair mechanisms [67,68].

Early clinical studies reported acceptable tolerability and supported further evaluation [69]. In the SYNERGY trial, opicinumab did not meet its primary composite disability endpoint in the overall population, although exploratory subgroup analyses suggested heterogeneity of response [70].

The AFFINITY trial evaluated opicinumab as an add-on therapy to DMTs over 72 weeks in relapsing MS. The primary endpoint, Overall Disability Response Score (ODRS), did not differ significantly between treatment and placebo groups [71]. The absence of consistent clinical benefit may reflect the biological heterogeneity of MS lesions, including variability in the availability of responsive oligodendrocyte precursor cells within chronic demyelinated plaques, as well as methodological challenges in selecting outcome measures sensitive to remyelination.

Opicinumab represents a biologically targeted remyelination strategy with a defined molecular rationale; however, clinical trials have not demonstrated consistent efficacy in unselected MS populations.

The principal mechanistic targets, translational signals, and clinical findings of adjunctive non-DMT strategies discussed in this review are summarized in Table 1.

## 5. Materials and Methods

This narrative review summarizes experimental, translational, and clinical studies addressing adjunctive non-DMT approaches in MS, with emphasis on immunometabolic modulation and remyelination-oriented strategies. A literature search was conducted using the PubMed database to identify relevant original articles, clinical trials, and reviews published up to January 2026. The search strategy included combinations of keywords reflecting the main thematic domains of the review, including “multiple sclerosis”, “adjunctive therapy”, “immunometabolism”, “remyelination”, and selected pharmacological agents such as “metformin”, “statins”, “PPAR agonists”, “curcumin”, and “opicinumab”.

To enhance transparency and reproducibility, the literature search was supplemented by manual screening of reference lists from relevant articles and review papers. Studies were selected based on relevance to the scope of the review, including mechanistic insights, preclinical evidence, and clinical outcomes relevant to adjunctive non-DMT strategies in MS.

Priority was given to studies providing translational linkage between molecular mechanisms and clinical observations, as well as to clinical trials and systematic reviews where available. Both experimental and clinical studies were included to reflect the current state of evidence across different stages of investigation.

Exclusion criteria comprised studies not directly related to multiple sclerosis, articles lacking mechanistic or clinical relevance to adjunctive therapies, and publications with insufficient methodological detail. Given the narrative nature of the review, formal quantitative quality assessment and meta-analytic methods were not applied; however, emphasis was placed on critically appraising the consistency and translational relevance of the available evidence.

## 6. Challenges and Future Directions

Despite growing interest in adjunctive non-DMT strategies in MS, several challenges limit their translation from experimental observations to clinical application. A consistent finding across multiple therapeutic classes discussed in this review is the discrepancy between mechanistic plausibility and clinical efficacy. Many agents demonstrate immunomodulatory, metabolic, or remyelination-promoting effects in preclinical models, yet these biological signals are not consistently reflected in clinical outcomes such as disability progression or relapse reduction. This translational gap represents a central limitation in the development of adjunctive therapies [4,21,46].

One important contributing factor is the biological heterogeneity of MS. Disease stage-dependent differences in inflammatory activity, neurodegeneration, and remyelination capacity may influence therapeutic responsiveness. Interventions targeting immunometabolic pathways or oligodendrocyte differentiation may therefore exert context-dependent effects that are not captured in heterogeneous, unselected clinical trial populations [12,70]. This highlights the need for biologically informed patient stratification, incorporating disease phenotype, stage, and underlying metabolic status.

Another key challenge relates to the selection of appropriate clinical endpoints. Conventional outcomes, such as relapse rate or global disability scales, may not adequately reflect processes targeted by adjunctive therapies, including remyelination or neuroprotection. The development and validation of more sensitive outcome measures—such as advanced neuroimaging markers, electrophysiological assessments, and fluid biomarkers—may be necessary to detect biologically relevant treatment effects [51,59].

The interaction between systemic metabolic factors and CNS pathology represents an additional layer of complexity. Emerging evidence suggests that metabolic comorbidities, including obesity, dyslipidemia, and insulin resistance, may influence both disease progression and treatment response in MS [10,11]. In this context, agents with immunometabolic activity may exert differential effects depending on the systemic metabolic environment, further complicating the interpretation of clinical trial results.

Furthermore, most available clinical studies evaluating adjunctive therapies are limited by small sample sizes, short follow-up periods, and heterogeneity in study design. The lack of adequately powered randomized controlled trials restricts the ability to draw definitive conclusions regarding clinical efficacy [33,43]. In addition, variability in treatment duration and concomitant DMT use may further confound the interpretation of results.

Future research should focus on addressing these limitations through the design of biologically informed clinical studies. Stratified trial designs targeting specific patient subgroups, including those defined by metabolic profiles or disease stage, may improve the ability to detect clinically meaningful effects. Integration of mechanistic biomarkers into clinical trials may further facilitate understanding of treatment responses and help bridge the gap between experimental and clinical findings [8,30].

Another promising direction involves combination strategies integrating DMTs with adjunctive agents targeting complementary pathways, such as immunometabolism and remyelination, with the aim of addressing multiple components of MS pathophysiology simultaneously [66,71].

In summary, while adjunctive non-DMT strategies offer a biologically plausible approach to targeting processes not fully addressed by current therapies, their clinical utility remains uncertain. Progress in this field will depend on improved alignment between mechanistic rationale, patient selection, and outcome assessment in future studies.

## 7. Conclusions

Adjunctive non-DMT approaches in multiple sclerosis aim to address biological processes extending beyond inflammatory disease activity. Immunometabolic modulators, including statins, metformin, and PPAR agonists, target cellular energy regulation, lipid metabolism, and inflammatory signaling pathways implicated in chronic neuroinflammation and tissue injury. Although experimental studies provide a mechanistic rationale for these interventions, clinical evidence remains heterogeneous and has not consistently demonstrated meaningful effects on disability progression or long-term clinical outcomes.

Remyelination-oriented strategies focus on promoting oligodendrocyte differentiation and myelin repair. Agents such as clemastine fumarate and opicinumab illustrate the translational challenges associated with targeting remyelination in MS. Despite clearly defined molecular targets and supportive experimental findings, clinical trials have so far shown modest or inconsistent therapeutic signals in unselected patient populations.

In parallel, nutraceutical and bioactive compounds, including polyphenols and plant-derived molecules, have been investigated because of their immunomodulatory, antioxidant, and metabolic effects. While these compounds demonstrate biological activity in experimental models and small translational studies, robust clinical evidence supporting disease-modifying benefits remains limited.

Overall, adjunctive therapies targeting immunometabolic pathways or remyelination processes remain investigational. Future research should prioritize biologically informed patient stratification, improved endpoint selection reflecting repair and neuroprotection, and the integration of mechanistic biomarkers to better define the potential role of these approaches alongside established disease-modifying therapies.

## Figures and Tables

**Table 1:** Adjunctive Non-Disease-Modifying Therapeutic Strategies in Multiple Sclerosis.

Substance	Mechanism	Reported Translational Signals	Clinical Evidence Summary	Key References
Clemastine fumarate	M1 muscarinic receptor antagonism; promotion of OPC differentiation	Reduced VEP latency in chronic optic neuropathy; experimental evidence of enhanced oligodendrocyte differentiation and remyelination in demyelination models	Modest electrophysiological signal; no significant effect on relapse rate or disability progression	[59,62,63,64,66,72]
Opicinumab (anti-LINGO-1)	LINGO-1 inhibition within the Nogo receptor signaling complex; enhanced oligodendrocyte differentiation and remyelination	Exploratory subgroup signals in phase II trials; preclinical evidence of improved remyelination and axonal conduction following LINGO-1 blockade	No significant difference in primary composite disability endpoints (SYNERGY, AFFINITY)	[67,68,69,70,71]
Statins	Th1 → Th2 shift; LFA-1 inhibition; oligodendroglial modulation	Reduced annualized brain atrophy rate (SPMS); immunomodulatory effects in EAE	Inconsistent effects on disability progression; no consistent disease-modifying efficacy	[4,12,17,22,23,25]
Metformin	AMPK activation; reduced NF-κB signaling; modulation of microglia and OPC differentiation	Reduced EAE severity; enhanced remyelination in aging models; modulation of metabolic and inflammatory biomarkers	No consistent reduction in relapse rate or disability progression; biological activity demonstrated	[8,28,29,32,33,35,36]
PPAR agonists (including fibrates)	PPAR-α/γ activation; transcriptional regulation of inflammatory and metabolic genes	Reduced pro-inflammatory cytokines in experimental models	Insufficient clinical evidence; no robust MS trials demonstrating efficacy	[20,21,43,44,45]
Epigallocatechin-3-gallate (EGCG)	Antioxidant; modulation of inflammatory and metabolic signaling pathways	Changes in inflammatory and metabolic markers; OCT changes in progressive MS	Heterogeneous results; no consistent effect on disability progression	[46,48,50,51,52]
Herbal compounds (multi-component formulations)	NF-κB and MAPK modulation; mitochondrial regulation	Reduced inflammatory mediator production in experimental settings	Limited, small, heterogeneous clinical studies; insufficient evidence for clinical efficacy	[53,54,58]
Curcumin	Modulation of NF-κB, PI3K/Akt/mTOR, AMPK and PPAR signaling; antioxidant and immunometabolic regulation	Reduced neuroinflammation and demyelination in EAE models; modulation of cytokine signaling and oxidative stress pathways	Limited clinical evidence; small studies suggest modulation of inflammatory markers, but no consistent effects on relapse activity or disability progression	[55,56,57]

Abbreviations: MS—multiple sclerosis; AMPK—adenosine monophosphate-activated protein kinase; NF-κB—nuclear factor-κB; OPC—oligodendrocyte precursor cell; EAE—experimental autoimmune encephalomyelitis; SPMS—secondary progressive multiple sclerosis; VEP—visual evoked potential; OCT—optical coherence tomography; PPAR—peroxisome proliferator-activated receptor; LFA-1—leukocyte function antigen-1.

## Data Availability

No new data were created or analyzed in this study. Data sharing is not applicable to this article.
